# Prevalence of dyslipidaemia within Polish nurses. Cross-sectional study - single and multiple linear regression models and ROC analysis

**DOI:** 10.1186/s12889-024-18542-6

**Published:** 2024-04-10

**Authors:** Anna Bartosiewicz, Justyna Wyszyńska, Piotr Matłosz, Edyta Łuszczki, Łukasz Oleksy, Artur Stolarczyk

**Affiliations:** 1 Institute of Health Sciences, Medical College of Rzeszów University, Rejtana 16 C, 35-959 Rzeszów, Poland; 2 Institute of Physical Culture Sciences, Medical College of Rzeszów University, 35-959 Rzeszów, Poland; 3 Department of Physiotherapy, Faculty of Health Sciences, Jagiellonian University Medical College, 31-007 Kraków, Poland; 4 Department of Orthopedics and Rehabilitation, Medical University of Warsaw, 04-749 Warsaw, Poland

**Keywords:** Nurses, Dislipidaemia, Metabolic disorders, Risk factors

## Abstract

**Background:**

Maintaining good health is vital not only for own well-being, but also to ensure high-quality patient care. The aim of this study was to evaluate the prevalence of dyslipidaemia and to determine the factors responsible for the development of this disorder among Polish nurses. Lipid profile disorders are the most prevalent and challenging risk factors for the development of cardiovascular disease. Nurses have significant potential and play a crucial role in providing care and treatment services.

**Methods:**

This cross-sectional study involved nurses and included measurements of body weight composition (Tanita MC-980), body mass index, waist circumference, blood pressure (Welch Allyn 4200B), lipid profile, and fasting blood glucose (CardioChek PA).

**Results:**

The results revealed that more than half of the nurses (60.09%) were overweight or obese, with 57.28% exhibiting elevated blood pressure, 32.25% having fasting glucose levels, and 69.14% experiencing dyslipidaemia. Multiple model evaluation using ROC curves demonstrated that multiple models accurately predicted hypercholesterolemia (AUC = 0.715), elevated LDL (AUC = 0.727), and elevated TC (AUC = 0.723) among Polish nurses.

**Conclusion:**

Comprehensive education programmes should be implemented that include the latest advances in cardiovascular disease prevention. Regular check-ups, as well as the promotion and availability of healthy food in hospital canteens, are essential.

**Supplementary Information:**

The online version contains supplementary material available at 10.1186/s12889-024-18542-6.

## Introduction

Nurses are the largest medical profession in the world, accounting for almost 60% of all medical workers in the global health sector. They have great potential and play a pivotal role in providing care and treatment services [1]. However, due to stress, overwork, high patient demands and workplace hazards, the incidence of morbidity among healthcare workers is higher compared to the general population [2, 3]. Long shift work hours, disturbance of the natural rhythm of sleep and metabolism combined with low physical activity predispose to the development of non-communicable diseases [4–8].

Lipid profile disorders are the most widespread and difficult to control risk factor predisposing to the development of cardiovascular disease, the most common cause of death in the world. among nurses [9–10]. In combination with smoking, low physical activity, overweight, and obesity, they are the main factors in the development of atherosclerosis and its complications [11, 12]. Taking into account data from the past five years, the life expectancy of Polish nurses is approximately 20 years less than that of the average Polish woman [13]. The median age at which nurses die is only 61.5 years, significantly less than the current median age of death for women in Poland as a whole, which is 81.8 years [13].

The data mentioned above are really alarming and indicate that the health conditions among nurses can be very poor. However, such data should be a trigger for further research on this issue. Subsequently, such research might be a basis for the implementation of non-communicable diseases prevention programmes among nurses. Prevention efforts should also address myocardial ischemia with no obstructive coronary arteries (INOCA), a chronic coronary syndrome that is recognized as a significant contributor to adverse cardiovascular outcomes, including myocardial infarction and heart failure with preserved ejection fraction. Although INOCA affects both women and men, women are more likely to be diagnosed with INOCA and experience more severe effects of angina, leading to recurrent hospitalisations and reduced quality of life. This is particularly relevant for nurses, as most of them are women [14].

This study is one of the first to do so comprehensively in Poland that evaluates parameters related to the prevalence of dyslipidaemia in this occupational group.

The purpose of the study was to evaluate the prevalence of dyslipidaemia and to determine the factors responsible for the development of this disorder among Polish nurses.

## Materials and methods

The study was carried out according to the Declaration of Helsinki guidelines and was approved by the University Bioethics Commission (Resolution No. 2022/088). Before the study was started, written informed consent was obtained from nurses. No information allows respondents to be identified.

### Study participants

The study was conducted in 2022 among nurses working in a randomly selected hospital in Rzeszów, after obtaining the hospital director’s consent to carry out the measurements among nurses. The project has also been approved by the head of nurses. In consultation with hospital authorities, the information included dates, hours, and the scope of the planned examinations for the attention of all nurses working on individual hospital wards. Nurses willing to participate in the study could sign up on the prepared list and choose a convenient date. All measurements (body mass analysis, glucose level, lipid profile) were performed in the morning from 7.00 to 10.00 a.m., at least 8 h of fasting. Participation in the study was voluntary and free. The following recruitment criteria were used: professionally active nurses, with no infection symptoms within the last 2 weeks, unaware of health problems, not undergoing treatment for the aforementioned conditions or medical nutritional therapy, and willing to participate in the project. Data from the measurements of 405 nurses were subjected to statistical analysis.

### Measurement

All measurements were carried out took place in the following order: signing an individual consent to participate in the study, 10 min of rest, completing a questionnaire, blood pressure measurement, glucose and lipid profile measurement, anthropometric measurements, and body composition analysis.

### Blood pressure (BP) measurement

Systolic blood pressure (SBP) and diastolic blood pressure (DBP) were measured after a 10 min rest in the sitting position with the back supported and the feet on the floor. Measurements were made in the right arm with the elbow placed at heart level using a cuff adjusted to the arm circumference of the subjects. A Welch Allyn 4200B apparatus (Aston Abbotts, UK) was used for the measurements. Measurements were made three times according to the guidelines of experts of the European Society of Hypertension [14]. The average of three measurements was calculated for each participant.

The following cutoff criteria were used:

- Normal blood pressure: 120–129 mmHg / 80–84 mmHg,
- Normal high pressure: 130–139 mmHg / 85–89 mmHg,
- Grade 1 hypertension: 140–159 mmHg / 90–99 mmHg,
- Grade 2 hypertension: 160–179 mmHg / 100–109 mmHg,
- Grade 3 hypertension: ≥ 180 mmHg / ≥ 110 mmHg [15].

### Body composition measurement

Body composition was measured by bioelectric impedance (6.25 kHz, 50 kHz, 90 µA) using a certified and calibrated analyzer (Tanita MC-980 PLUS MA, Tokyo, Japan) with an accuracy of 0.1 kg/0.1%. The analyzer has 8 electrodes (4 built in the platform and 4 in the holders). To ensure accurate measurements, the device was placed on a level surface according to the instructions, in such a way as to ensure that the level indicator is centered on the level meter. The participants entered the analyzer platform barefoot, in light clothing, in an upright position, motionless, placing their feet in a way that ensured an even distribution of body weight and contact with the electrodes. Measurement was carried out in a standing position, contact with the electrodes was provided by bare feet and hands held away from the body at an angle of 35°-40°. The Tanita software automatically measured body mass and impedance to determine the percentage of body fat, based on formulas and equations that are generalized to standard adults. The Tanita MC 980 device has approvals for medical use and meets the NAWI and CLASS III standards and the MDD 93/42/EEC directive, as well as the CE0122 EU certificate [16].

### Anthropometric measurement

Anthropometric measurements were measured using an anthropometric tape according to the ISAK protocol (The International Society of the Advancement of Kinanthropometry) by a member of the ISAK Level 3 accredited research team, a full profile anthropometrist technician. Body height was measured with an accuracy of 0.1 cm using a Seca 213 stadiometer. The measurements were made under standard conditions, barefoot in an upright position. The mean value of three measurements was used in the analyses.

### Waist hip ratio (WHR)

The following normative ranges were adopted for the value of the WHR index:

- Men:
- < 0.96 WHR within normal limits
- ≥ 0.96 Abdominal obesity, increased risk of metabolic diseases.
- Women:
- < 0.83 WHR within normal limits
- ≥ 0.83 Abdominal obesity, increased risk of metabolic diseases [17].

### Waist-to-height ratio (WHtR)

The following normative ranges were adopted for the value of the WHtR index:

- Men and women:
- < 0.5 value of the index within normal limits
- 0.5–0.6 increased cardiometabolic risk
- ≥ 0.6 significantly increased cardiometabolic risk [18].

### Body mass index (BMI)

BMI was calculated automatically by the Tanita MC-980 MA body composition analyzer Tanita MC-980 MA) as body weight (kg)/height (m)^2^. Criteria of body mass deficiency, normal body weight, overweight, and obesity recommended by the Centers for Disease Control and Prevention were used [19].

The following cut-off criteria of Body Mass Index were adopted:

- 17–18.49 = underweight,
- 18.5–24.99 = normal body weight,
- 25–29.99 = overweight,
- 30–34.99 = 1st degree.
- 35–39.99 = 2nd degree obesity,
- > 40 = 3rd degree obesity [19].

### Laboratory tests

A finger prick capillary whole blood sample was collected from each nurse and analysed using the CardioChek PA (CCPA) analyzer according to the manufacturer’s instructions. Capillary samples were collected by a registered nurse, observing all the rules of asepsis and antisepsis.

Using the collection device supplied with the CCPA that was compatible with the CardioChek reagent test strips (35–40 µL sample volume). Blood samples were collected in the morning after a fast of 9–12 h of the night. Total cholesterol (TC), low-density lipoprotein (LDL), high-density lipoprotein (HDL), triglycerides (TG), and glucose levels were evalueted. The CCPA device was checked each time before starting by Internal Quality Control using a control strip recommended by the manufacturer. Maintenance was performed according to the manufacturer’s instructions [20, 21].

### Dyslipidemia cut point criteria

Hypercholesterolemia was diagnosed when TC was ≥ 190 mg/dL or LDL > 115 mg/dL. Atherogenic dyslipidemia was determined when TG was ≥ 150 mg/dL, HDL < 40 mg/dL in males and < 48 mg/dL in females, and elevated LDL fraction (> 130 mg/dl).

Hypertriglyceridemia was determined when TG was > 150 mg/dL with normal LDL levels (< 115 mg/dl) and severe hypertriglyceridemia - TG ≥ 800 mg/dL [22, 23].

### Lipid profile cut point criteria

TC: 150–190 mg/dl.

LDL cholesterol: less than 115 mg/dl.

HDL cholesterol: men above 40 mg/dl, women over 48 mg/dl,

TRG: below 150 mg/dL [24].

### Fasting glucose cut point criteria

Less than 70 mg / dl - hypoglycemia,

70 to 99 mg/dL - normal glucose level,

100 to 125 mg/dL - elevated glucose levels - pre-diabetes,

≥ 126 mg/dL at least two measurements - diabetes mellitus [25].

Furthermore, based on the results of the lipid profile the atherogenicity indicators are determined, e.g. Castelli index (TC/HDL), LDL/HDL ratio, and TG/HDL ratio. Elevated values of these indicators demonstrate the risk of developing atherosclerosis, ischemic heart disease, or stroke, and indicate the need to immediately change eating habits and/or pharmacotherapy.

### Atherogenicity indicators cut points

TC/HDL: men: <4.5; women: <4.0,

LDL/HDL ratio - the correct result should be 1:3 − 1:2.

TG/HDL ratio: the optimal score is < 2, which means that we have mainly large LDL lipoprotein particles, and therefore harmless to our health. A score > 2 means a high risk of developing cardiovascular disease due to the predominance of mainly small and dense LDL particles [22, 26].

### Questionnaires

The questionnaire package was prepared in paper form with an attached envelope, allowing the completed questionnaires to be sealed, and thus ensuring the confidentiality of the responses provided. The survey included questions about the sociodemographic data of the respondents including age, gender, place of work, type of work, and educational level (basic, bachelor’s, and master’s). Additional questions were related to the most frequently consumed groups of food products, salt intake, participation in preventive examinations, weight control, smoking (the response ‘yes’ indicates individuals who smoked regularly in the past as well as those who currently smoke, the response ‘no’ indicates that the person does not currently smoke and has not smoked in the past), work system, self-assessment of health condition.

### Quality Control

To ensure the high quality of the research conducted, a research strategy was developed. A four-person team of university employees consisting of a nurse, dietician, physiotherapist, and full profile anthropometrist technician were responsible for supervising the measurements, directing study participants to the appropriate measurement stations, collecting questionnaires, and coding the obtained results. The research survey and raw measurement data have been double-checked to ensure the authenticity and accuracy of the data, including effective data logic checks, and editing checks. To protect the personal data of the participants and ensure the confidentiality of these data, each nurse was assigned as an individual ID number, developed according to a previously adopted algorithm, which was used during the measurements, results processing, processing, and statistical analysis of the data. The database was built using Excel and data was entered twice by two data managers to ensure accuracy and integration.

### Statistical analysis

The analysis was performed using R programme, version 4.2.1 [27]. The analysis of quantitative variables (i.e. expressed in numbers) was performed by calculating the average, standard deviation, median, and quartiles. The analysis of qualitative variables (i.e. not expressed in numbers) was performed by calculating the number and percentage of occurrences of each value. A single and multiple analysis of the influence of many variables on a binary variable (presence or absence of hypercholesterolemia) was performed using logistic regression. Results are presented as values of odds ratio (OR) parameters with a 95% confidence interval. The variables for the multiple analysis was selected on the basis of their significance in the one-factor analyses. The EPV (events per variable) index for the analysis was 8.7. The quality of the multiple models were assessed using the ROC (Receiver operating characteristic) curves and the areas under curve (AUC). The analysis adopted a significance level of 0.05. Thus, all p-values below 0.05 were interpreted as significant associations.

## Results

### Characteristics of the study group

In total, 405 nurses participated in the measurements, the vast majority of the participants (*n* = 380; 93.83%) were women. The average age of the respondents was approximately 48.5 years (SD ± 10.44). Detailed characteristics of the study group are presented in Table 1.

**Table 1:** General characteristics of the Study Group

Variable		Total (*n* = 405) n (%)
Sex *	Female	380 (93.8)
	Male	25 (6.1)
Age [years]	Average ± SD	48.4 (10.37)
	Median (quartiles)	51 (42–55)
	Range	22–79
Place of residence *	City	209 (51.6)
	Village	196 (48.4)
Type of work*	Staff management/administration	60 (14.8)
	Hospital ward	344 (85.1)
Work system*	One shift work	140 (34.5)
	Shift work and night duty	265 (65.4)
More than one job *	No	238 (58.7)
	Yes	167 (41.2)
Education*	Basic nursing education	131 (32.3)
	Bachelor	95 (23.4)
	Master degree	179 (44.2)
Participation in preventive examinations other than obligatory*	No	293 (72.3)
	Yes	112 (27.6)
Cigarettes smoking *	No	305 (75.3)
	Yes	100 (24.6)
Adding sugar to coffe/tea *	No	175 (43.2)
	Yes	230 (56.7)
Salting dishes*	I don’t use salt at all	6 (1.4)
	Rarely or never add salt to food	71 (17.5)
	I taste the food and add salt as needed	234 (57.7)
	I add salt to my food without trying it first	94 (23.2)
Weight self-control*	Every day	16 (3.9)
	Once a week	69 (17.0)
	Twice a week	46 (11.3)
	Once a month	100 (24.6)
	Hardly ever	72 (17.7)
	I do not check my weight regulary	102 (25.1)
Self-assessment of the material situation *	Very good	30 (7.4)
	Good	208 (51.3)
	Average	158 (39.0)
	Bad	9 (22.2)

Data presented as: * - *n* (%); SD– standard deviation

The declaration of daily consumption of specific food product groups is as follows: white bread 56.3%, dark bread 25.43%, fish and seafood 1.73%, red meat and sausages 19.26%, sour milk products 27.9%, cheese 25.19%, cottage cheese 22.22%, fruits and vegeTable 70.12%, sweets and salty snacks 27.41%, fast food products 4.69%. The exact frequency of consumption of selected groups of food products is presented in Table 2.

**Table 2:** Consumption frequency of specific group of products

Group of products	Frequency of consumption				
	I don’t eat *n* (%)	A few times a month *n* (%)	2–4 times a week *n* (%)	Ones a week *n* (%)	Every day *n* (%)
White bread	36 (8.8)	45 (11.1)	49 (12.1)	47 (11.6)	228 (56.3)
Wholemeal bread	66 (16.3)	69 (17.0)	88 (21.7)	79 (19.5)	103 (25.4)
Fishes and seafood	60 (14.8)	193 (47.6)	35 (8.6)	110 (27.1)	7 (1.7)
Red meat, ham, sausages	35 (8.6)	111 (27.4)	117 (28,8)	64 (15.8)	78 (19.2)
Sour milk products	44 (10.8)	50 (12.3)	119 (29.3)	79 (19.5)	113 (27.9)
Cheese	22 (5.4)	114 (28.1)	85 (20.9)	82 (20.2)	102 (25.1)
Cottage cheese	12 (2.9)	73 (18.0)	141 (34.8)	89 (21.9)	90 (22.2)
Vegetables/fruit	3 (0.7)	11 (2.7)	76 (18.7)	31 (7.6)	284 (70.1)
Sweets/salty snacks	13 (3.2)	65 (16.0)	132 (32.5)	84 (20.7)	111 (27.4)
Fast food products	168 (41.4)	159 (39.2)	28 (6.9)	31 (7.6)	19 (4.6)

The results of BMI, WHR, WHtR, blood glucose, body fat percentage (BFP), visceral fat index (VFI), total body water and phase angle are shown in Table 3.

**Table 3:** Body composition parameters, glucose levels and BP in the study group

Parameter		Total (*n* = 405) n (%)
BMI * [kg/m^2^]	Underweight	7 (1.73)
	Normal body mass	158 (39.01)
	Overweight	132 (32.59)
	Class I obesity	74 (18.27)
	Class II obesity	25 (6.17)
	Class III obesity	9 (2.22)
WHR*	Normal	208 (51.36)
	Abdominal obesity	197 (48.64)
WHtR *	Normal	178 (43.95)
	Increased cardiometabolic risk	164 (40.49)
	Significantly increased cardiometabolic risk	63 (15.56)
BP* [mmHg]	Normal	173 (42.72)
	Elevated	71 (17.53)
	High blood pressure Stage 1	126 (31.11)
	High blood pressure Stage 2	35 (8.64)
FG* [mg/dL]	Hypoglicemia Normal	2 (0.49) 274 (67.65)
	Elevated	123 (30.37)
	Abnormal glucose - suspicion of diabetes	6 (1.48)
BFP category * [%]	Normal	233 (57.53)
	Elevated	123 (30.37)
	Abnormal glucose - suspicion of diabetes	49 (12.10)
VFI *	Normal	386 (95.31)
	Elevated	19 (4.69)
TBW * [%]	Normal	284 (70.12)
	Low	106 (26.17)
	High	15 (3.70)
PA [°]	Average ± SD	5,51 (0.5)
	Median (quartiles)	5,47 (5.19–5.8)
	Range	3.9–7.66

Data presented as: * - n (%); BMI- Body Mass Index; WHR - Waist Hip Ratio; WHtR - Waist to Height Ratio; BP - Blood pressure; FG - Fasting glucose; BFP category - Body Fat Percentage category; VFI - Visceral Fat Index; TBW - Total Body Water; PA– Phase angle; SD– standard deviation

The results showed that more than half of the nurses participating in the measurements (*n* = 249; 61.48%) had hypercholesterolemia. Severe hypertriglyceridemia has not been reported. The occurrence of individual forms of dyslipidemia in the study group is presented in Table 4.

**Table 4:** Prevalence of dyslipidaemia and its forms in the study group

A type of dyslipidaemia	*n*	% *
Hypercholesterolaemia	249	61.48
Atherogenic dyslipidaemia	32	7.90
Hypertriglyceridaemia	50	12.35
Severe hypertriglyceridaemia	0	0.00
Dyslipidaemia	280	69.14
Increased Castelli Index	114	28.15
Increased LDL/HDL ratio	65	16.05
Increased TG/HDL ratio	198	48.89

* The value does not add up to 100, as multiple choice was possible. LDL - low-density lipoprotein cholesterol; HDL - high-density lipoprotein cholesterol; TG– triglycerides

Single-factor logistic regression models (separate for each of the analyzed features) showed that significant predictors (p<0.05) of the risk of developing hypercholesterolemia are age (OR = 0.058), participation in preventive examinations other than obligatory (OR = 1.834), obesity and BMI grade (OR = 3.096), WHR abdominal obesity (OR = 2.135), WHtR increased cardiometabolic risk (OR = 1.969), WHtR significantly increased cardiometabolic risk (OR = 2.117), SBP (OR = 1.012), hypertension Stage I (OR = 1.888), stage II hypertension (OR = 15.573), increased body fat (OR = 1.641), excessive body fat (OR = 3.199), low body water (OR = 2.085). The multiple logistic regression model showed that age (OR = 1.038) and grade II hypertension (OR = 12.589) are significant (p<0.05) independent predictors of the risk of developing hypercholesterolemia (details available in supplementary materials **-** Table S1).

**Fig. 1:**
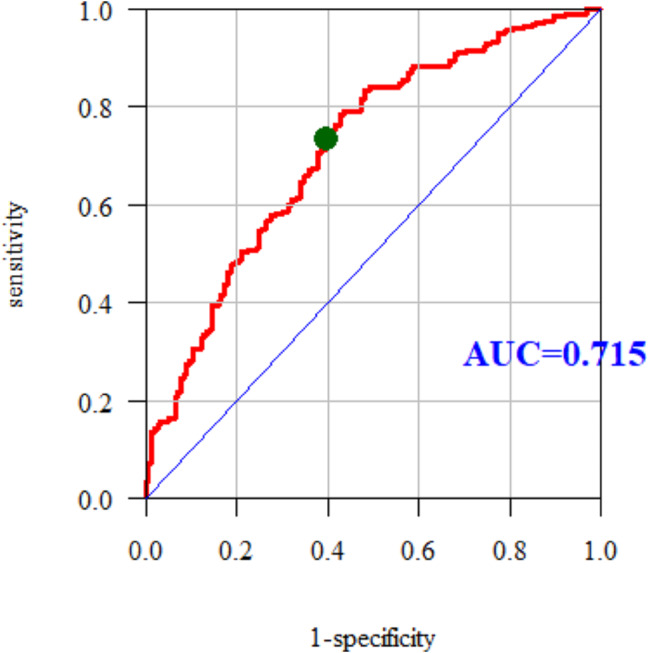
ROC curve and AUC value for hypercholesterolaemia

The quality of the multidimensional models was assessed using the receiver operating characteristic (ROC) curves and the areas below them. The area under curve (AUC) for the multiple models was 0.715. Therefore, the multiple model predicts the occurrence of hypercholesterolemia quite well (Fig. 1).

Single-factor logistic regression models (separate for each of the analyzed features) showed that significant (p<0.05) predictors of the risk of developing elevated LDL are: age (OR = 1.052), higher education (OR = 0.572), participation in preventive examinations other than obligatory (OR = 1.999), consumption of fish and seafood several times a month (OR = 0.517), consumption of fast food products (OR = 0.584), 1st degree obesity according to BMI (OR = 2.595), abdominal obesity according to WHR (OR = 2.354), increased cardiometabolic risk by WHtR (OR = 2.078), significantly increased cardiometabolic risk by WHtR (OR = 2.392), SBP (OR = 1.012), grade I hypertension (OR = 2.405), grade II hypertension (OR = 2.354), = 6.328), excessive body fat (OR = 3.435) and low body water content (OR = 2.299). The multiple logistic regression model showed that significant independent predictors (p<0.05) of the risk of developing elevated LDL are: participation in prophylactic examinations other than obligatory (OR = 1.792), consumption of fish and seafood several times a month (OR = 0.514), consumption of fast food products (OR = 0.59), abdominal obesity according to WHR (OR = 1.748), 1st degree hypertension (OR = 2.632), 2nd degree hypertension (OR = 7.096), (details available in supplementary materials - Table S2).

**Fig. 2:**
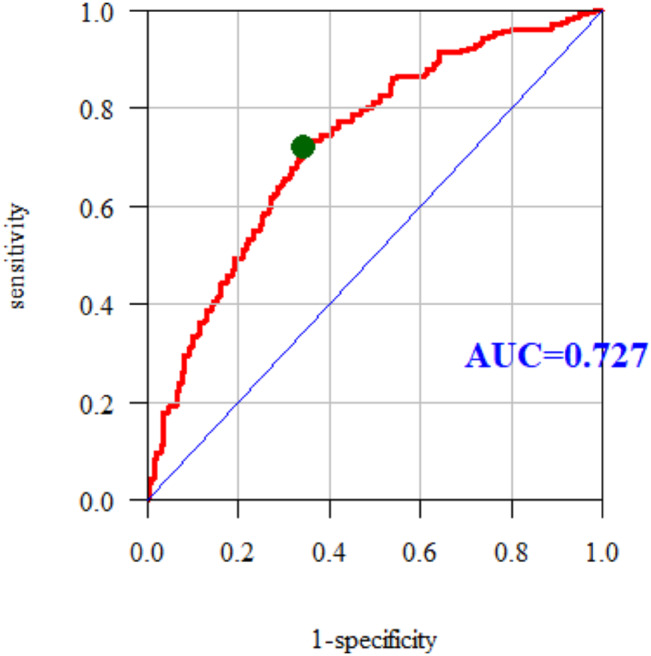
ROC curve and AUC value for LDL

The area under curve (AUC) for the multiple models was 0.727. The multiple model predicts the occurrence of an elevated low-density lipoprotein (LDL) level in the study group quite well (Fig. 2).

Logistic regression models (separate for each of the analyzed characteristics) showed that significant (p<0.05) predictors of the risk of developing elevated TC are: male gender (OR = 0.363), age (OR = 1.061), participation in preventive examinations other than obligatory (OR = 1.855), 1st degree obesity according to BMI (OR = 2.595), abdominal obesity according to WHR (OR = 2.047), increased cardiometabolic risk according to WHtR (OR = 1.895), SBP (OR = 1.012), 1st degree hypertension ( OR = 1.864), grade II hypertension (OR = 11.043), excessive body fat (OR = 2.352) and low body water (OR = 1.853). The multiple logistic regression model showed that significant (p<0.05) independent predictors of the risk of elevated TC are male gender (OR = 0.279), age (OR = 1.042) and grade II hypertension: OR = 11.487), (details available in supplementary materials - table S3).

**Fig. 3:**
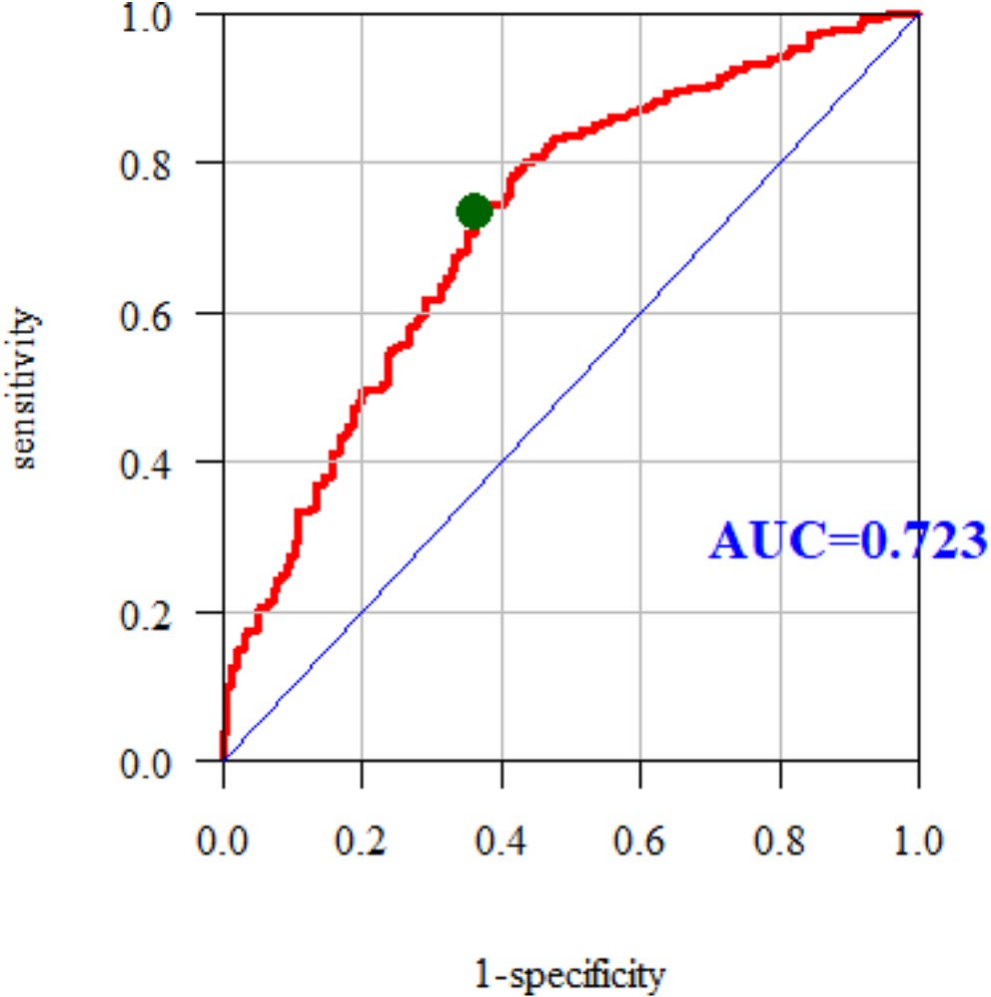
ROC curve and AUC value for TC

The area under curve (AUC) for the multiple models was 0.723. Therefore, the multiple model predicts the occurrence of an elevated total cholesterol (TC) level quite well (Fig. 3).

## Discussion

To our knowledge, this is the first study to evaluate the prevalence of individual components of dyslipidaemia and to determine the factors responsible for the development of this disorder in the nursing population. Most of the nurses who participated in the measurements had lipid profile disorders (*N* = 280; 69.14%), elevated blood pressure (*N* = 232; 57.28%), increased body weight (*N* = 231; 57.03%) and 31% had elevated glucose levels.

The purpose of the study was to evaluate the prevalence of dyslipidaemia and to determine the factors responsible for the development of this disorder among Polish nurses.

Most studies are related to patients, but few assess the health of healthcare professionals providing comprehensive health care [2]. According to the WHO, only half of countries in the world have health profiles of their employees, unfortunately, noncommunicable diseases and those related to lifestyle are not taken into account to the least extent [28]. Due to the overload of work and the growing demands of patients and employers, healthcare professionals pay little attention to their personal well-being and health [29]. Taking into account the data of the last five years, the median age of death for nurses is only 61.5 years, which is much lower than the current median age of death for Polish women overall, which is 81.8 years [13]. The study conducted on a population of thousands of people over 15 years has shown that the health condition of healthcare workers is much worse compared to the general population. Researchers based on large national medical history data sets have also shown that the percentage of deaths among physicians and nurses is much higher [30]. The specificity of the work environment of nurses is the cause of physical and biological hazards, violence, and loads on the musculoskeletal system. Furthermore, shift work and the resulting disturbances in the natural rhythm of sleep, rest, regular meals, and lack of time for physical activity predispose to the development of many metabolic diseases [31–33].

Our research has shown that more than half (56.3%) of nurses declared daily consumption of white bread. Sweets/salty snacks and cheese are eaten daily by more than a quarter of nurses, and red meat and its products, as well as fast food products, are eaten by almost 20% of the nurses surveyed. Furthermore, the respondents declare a relatively low consumption of fish and seafood; only a small percentage of the respondents (0.74%) do not include fruits and vegetables in their daily diet. The insufficient consumption of these products is indicated by the results of a study conducted among nurses in Delhi [34]. Results Nora et al. showed that the factor that affected the consumption of certain product groups and the deterioration of eating habits was stress at work or work under onerous circumstances. According to the researchers, nurses who worked with COVID-19 patients ate significantly less fruit and vegetables. Additionally, up to 76.2% of nurses declared financial difficulties in buying healthy food [35]. In another study, most nurses indicated that it is difficult to maintain proper eating habits at work, because the food they often receive from patients as a gift contains a lot of sugar and fat and is easily available [36, 37]. In the study by Wojcza et al. almost all nurses (94.5%) included in the survey declared bad eating habits, especially nurses who were overweight/obese and declared additional employment [38].

Our measurements showed that more than half of nurses (60.09%) were overweight and/or obese. Furthermore, the values of the WHR and WHtR indices in almost half of the subjects indicated the presence of abdominal obesity and increased cardiometabolic risk. The results of measurements made using a body composition analyser showed that 42.47% had increased or excessive body fat. Many studies confirm that the occurrence of overweight and obesity among nurses is quite common. A Scottish cross-sectional study showed that obesity among nurses is more common not only in relation to the general population, but also among other healthcare professionals [39, 40]. A similar trend is also observed in Great Britain, New Zealand, the United States, and Australia [41, 42]. In a study by Kayaroganam et al., evaluating the profiles of noncommunicable disease risk factors among nurses in a tertiary care hospital in South India, more than two-thirds of nurses had abdominal obesity (71.6%), more than half were obese (57.2%), and one fifth were overweight (20.5%), mainly women over 40 years of age [12].

In our study, a total of 57.28% of nurses had elevated blood pressure and hypertension of the first and second degree. Furthermore, a total of 32.25% of the subjects had glucose abnormalities, most of which were related to elevated glucose levels. Hypertension is a global problem and the leading cause of morbidity and premature death [43]. In the Manakali study, 52% of nurses had hypertension, significantly higher than the reported prevalence of hypertension among nurses in South Africa (20%), Brazil (32%) and healthcare professionals in Nigeria (20.1%). This result is also higher than the documented prevalence of hypertension in the general adult population of South Africa [44–47]. In the Kayaroganam study, about a quarter of nurses had elevated BP (14.4%) and elevated blood glucose (11.5%) [39]. Some studies indicate that elevated glucose levels among nurses may also be the result of stress experienced at their daily work [48, 49].

Dyslipidaemia occurred in 69.14% of nurses who participated in the measurements and was most often related to hypercholesterolemia (61.48%). There was also an increased level of atherogenicity indexes, especially in the case of the TG/HDL index. Dyslipidaemia predisposes to the development of cardiovascular diseases, atherosclerosis, and, consequently, to ischemic heart disease and stroke [50]. The results of a study conducted in South India showed that more than one-third of nurses had hypercholesterolemia (34.3%) and elevated LDL (41.9%) and two-thirds had low HDL (65.3%). Hypercholesterolemia and triglyceridemia were significantly higher among men and those who were married. Elevated LDL levels were significantly higher among men and people < 50 years of age, while low HDL levels were significantly higher among women 30 to 39 years of age [39]. Researchers from Ghana observed that an increase in glycemia was associated with a corresponding increase in atherogenic lipid parameters. Among nurses with atherogenic dyslipidaemia (26.29%), there were also numerous lipid abnormalities. A significant additive trend was observed between the mean markers of atherogenic dyslipidaemia and the degree of obesity. In all cases, the levels of atherogenic markers of dyslipidaemia increased from underweight and reached peak at overweight or obesity [51]. Regression analysis showed that significant predictors (*p* < 0.05) of the risk of developing hypercholesterolemia in the one-factor model are age, participation in preventive examinations other than obligatory, obesity of the first degree, abdominal obesity, increased and significantly increased cardiometabolic risk, SBP, hypertension of the first and second degree, increased and excessive body fat and low body water content. In the case of the risk of developing elevated LDL, education, rare consumption of fish and seafood, and consumption of fast-food products are also important predictors, while in the case of elevated TC, male gender. Significant independent predictors (p<0.05) of the risk of hypercholesterolemia in the multiple models are age and grade II hypertension, in the case of LDL, in addition to those listed, participation in preventive examinations, rare fish consumption, fast food consumption, abdominal obesity and grade I and II hypertension. However, in the case of the risk of elevated TC, male sex, age, and grade II hypertension. Hojat et al. analysing risk factors for cardiovascular diseases among male and female nurses showed that there was a statistically significant difference between women and men in indicators such as eating breakfast, family history, fruit consumption, high-density lipoprotein (HDL), BMI, and WHR [52]. Whereas in study by Gadallah et al., the only predictor of high LDL was age ≥ 40 years, whereas unhealthy diet and night shift work were predictors of risky HDL levels [53].

The nature of nurses’ work predisposes them to many health problems. Shift work and disrupting the natural rhythm of sleep, rest and regularity of eating meals are the cause of overweight, obesity, hypertension, dyslipidaemia, and, consequently, cardiovascular diseases. A study by the Cardiovascular Nurses Association showed that hypertension, heart disease, and dyslipidaemia are common among nurses [54]. Health professionals are an important resource for each country [55]. Taking care of your health, you can be good models for your patients, families, and friends [56]. The Galen, one of the most eminent ancient physicians, has said: “The physician will hardly be thought very careful of the health of his patients if he neglects his own”. It should be taken into account for creating solutions at the government and institutional level, limiting risk factors for progression dyslipidaemia among nurses [2].

### Strengths, limitations, and future research

To our knowledge, this is one of the first such comprehensive studies in Poland to assess parameters related to the prevalence of dyslipidaemia in this occupational group. There are also several potential study limitations that should be considered when interpreting the results. This study had a limited geographic scope and should be extended to more medical facilities in other regions. Since the study is cross-sectional, causality and temporality issues should not be considered. More research is needed in a larger population across all age groups.

## Conclusions

The study showed that the prevalence of disorders of the lipid profile in the study group is high, especially hypercholesterolemia. Nurses declared bad eating habits, which could be the cause of obesity, overweight, elevated glucose levels, and hypertension. The evaluation of multiple models using ROC curves showed that the multiple models predict hypercholesterolemia (AUC = 0.715), elevated LDL (AUC = 0.727) and elevated TC (AUC = 0.723) quite well among the nurses surveyed. Our study shows most of the risk factors among the nursing population in the Subcarpathian region of Poland. The possession of medical knowledge and access to medical entities do not protect nurses against the risk of diseases. Broadly understood education should be implemented, including the latest trends in the prevention of cardiovascular disease. Regular check-ups and the promotion and availability of healthy food in hospital canteens are necessary.

### The practical implications for non-communicable disease prevention

Bearing in mind obtained results and literature review as well as considering the importance of prevention non-communicable diseases among nurses, the following recommendations could be included:

- Dietary modifications may involve recommendations to replace salt with potassium chloride and herbs, as well as eliminating the consumption of high-calorie and highly processed products. Particularly recommended are the Dietary Approaches to Stop Hypertension (DASH) diet for the prevention and treatment of hypertension, and the Mediterranean diet, which presents not only a way of nutrition but also a holistic model of a healthy lifestyle [57–59].
- Another important issue for improvement of nurses’ health conditions is engaging in physical activity beyond the scope of daily nursing duties. Here, with the support of employers, it is worth promoting exercise programs that encourage and facilitate physical activity [60, 61].
- For nurses at increased cardiovascular risk, it is crucial to consider the potential benefits of statin therapy, as recommended by reputable sources for individuals with elevated cardiovascular risk. This is particularly important for individuals for whom lifestyle modifications are challenging or ineffective. Offering guidance on the appropriate use of statins could assist in preventing cardiovascular diseases among nurses [62].
- It is also important to leverage expertise from multiple stakeholders in order to drive transformational change in building safe, secure and responsive healthcare working environments [63]. By incorporating these recommendations into the documents, can provide comprehensive guidance on health preventive among nurses, addressing the reviewer’s feedback effectively. The present study emphasizes on behavior change campaign among health professionals to motivate them to adopt healthy lifestyle and prevention of non-communicable diseases. Moreover, nurses who know their risk factors and who follow healthy lifestyle behaviors will be more effective in counseling roles for patients.

### Electronic supplementary material

Below is the link to the electronic supplementary material.

Supplementary Material 1

Supplementary Material 2

Supplementary Material 3

## Data Availability

The dataset supporting the conclusions of this article is included within the article (and its additional file).
