# Correction: Prevalence of dyslipidaemia within Polish nurses. Cross-sectional study - single and multiple linear regression models and ROC analysis

**DOI:** 10.1186/s12889-024-18776-4

**Published:** 2024-05-10

**Authors:** Anna Bartosiewicz, Justyna Wyszyńska, Piotr Matłosz, Edyta Łuszczki, Łukasz Oleksy, Artur Stolarczyk

**Affiliations:** 1 Institute of Health Sciences, Medical College of Rzeszów University, Rejtana 16 C, Rzeszów, 35-959 Poland; 2 Institute of Physical Culture Sciences, Medical College of Rzeszów University, Rzeszów, 35-959 Poland; 3 Department of Physiotherapy, Faculty of Health Sciences, Jagiellonian University Medical College, Kraków, 31-007 Poland; 4 Department of Orthopedics and Rehabilitation, Medical University of Warsaw, Warsaw, 04-749 Poland

**Correction: BMC Public Health (2024) 24:1002**

10.1186/s12889-024-18542-6

The original publication of this article contained an incorrect age range in table 1. The incorrect and correct information is listed in this correction article, the full table is shown in the original publication. The original article has been updated.

Incorrect

- 18-79

Correct

- 22-79

