# Supplementary material for: Prevalence of dyslipidaemia within Polish nurses. Cross-sectional study - single and multiple linear regression models and ROC analysis
Source: BMC Public Health. 2024 Apr 10;24:1002. doi: 10.1186/s12889-024-18542-6 (PMC11008020; doi:10.1186/s12889-024-18542-6)
Supplement: Supplementary file 1 — Supplementary Material 1 [file 12889_2024_18542_MOESM1_ESM.docx]

Table S2 presents single and multiple logistic regression models indicating significant (p < 0.05) predictors of the risk of developing elevated low-density lipoprotein (LDL) in the study group. Single-factor logistic regression models (separate for each of the analyzed features) showed that significant (p˂0.05) predictors of the risk of developing elevated LDL are: age (OR=1.052), higher education (OR=0.572), participation in preventive examinations other than obligatory (OR=1.999), consumption of fish and seafood several times a month (OR=0.517), consumption of fast food products (OR=0.584), 1st degree obesity according to BMI (OR=2.595), abdominal obesity according to WHR (OR=2.354), increased cardiometabolic risk by WHtR (OR=2.078), significantly increased cardiometabolic risk by WHtR (OR=2.392), SBP (OR=1.012), grade I hypertension (OR=2.405), grade II hypertension (OR=2.354), =6.328), excessive body fat (OR=3.435) and low body water content (OR=2.299). The multiple logistic regression model showed that significant independent predictors (p˂0.05) of the risk of developing elevated LDL are: participation in prophylactic examinations other than obligatory (OR=1.792), consumption of fish and seafood several times a month (OR=0.514) consumption of fast food products (OR=0.59), abdominal obesity according to WHR (OR=1.748), 1st degree hypertension (OR=2.632), 2nd degree hypertension (OR=7.096).

## Table S2. Risk factors for elevated low-density lipoprotein. Single and multiple analysis

| **Variable** | | **Univariate model** | | | | **Multiple model** | | | |
| --- | --- | --- | --- | --- | --- | --- | --- | --- | --- |
|  |  | **OR** | **95%CI** | | ***p*** | **OR** | **95%CI** | | ***p*** |
| Sex | Female | 1 | ref. |  |  |  |  |  |  |
|  | Male | 0.738 | 0.327 | 1.666 | 0.464 |  |  |  |  |
| Age | [years] | 1.052 | 1.03 | 1.073 | <0.001 * | 1.012 | 0.986 | 1.039 | 0.367 |
| Place of work | City | 1 | ref. |  |  |  |  |  |  |
|  | Village | 1.31 | 0.887 | 1.937 | 0.175 |  |  |  |  |
| Type of work | Staff management/administration | 1 | ref. |  |  |  |  |  |  |
|  | Hospital ward | 1.139 | 0.658 | 1.972 | 0.641 |  |  |  |  |
| Work system | One shift work [8h] | 1 | ref. |  |  |  |  |  |  |
|  | Shift work and night duty [12h] | 1.025 | 0.68 | 1.543 | 0.908 |  |  |  |  |
| More than one job | No | 1 | ref. |  |  |  |  |  |  |
|  | Yes | 1.484 | 0.997 | 2.21 | 0.052 |  |  |  |  |
| Education | Basic nursing education | 1 | ref. |  |  | 1 | ref. |  |  |
|  | Bachelor | 0.875 | 0.514 | 1.491 | 0.624 | 0.946 | 0.522 | 1.712 | 0.854 |
|  | Master degree | 0.572 | 0.363 | 0.902 | 0.016 * | 0.84 | 0.49 | 1.442 | 0.528 |
| Participation in preventive examinations other than obligatory * | No | 1 | ref. |  |  | 1 | ref. |  |  |
|  | Yes | 1.999 | 1.278 | 3.128 | 0.002 * | 1.792 | 1.092 | 2.94 | 0.021 * |
| Cigarettes smoking | No | 1 | ref. |  |  |  |  |  |  |
|  | Yes | 1.298 | 0.824 | 2.044 | 0.26 |  |  |  |  |
| Adding sugar to coffe/tea | No | 1 | ref. |  |  |  |  |  |  |
|  | Yes | 1.35 | 0..91 | 2.003 | 0.136 |  |  |  |  |
| Salting dishes | Rarely or never add salt to food | 1 | ref. |  |  |  |  |  |  |
|  | I taste the food and add salt as needed | 0.878 | 0.524 | 1.471 | 0.621 |  |  |  |  |
|  | I add salt to my food without trying it first | 0.956 | 0.523 | 1.748 | 0.884 |  |  |  |  |
| Weight self-control | Once a week or often | 1 | ref. |  |  |  |  |  |  |
|  | Twice a week | 0.955 | 0.463 | 1.971 | 0.901 |  |  |  |  |
|  | Once a month | 0.706 | 0.394 | 1.263 | 0.241 |  |  |  |  |
|  | Hardly ever | 0.622 | 0.33 | 1.17 | 0.141 |  |  |  |  |
|  | I do not check my weight regulary | 0.706 | 0.396 | 1.261 | 0.24 |  |  |  |  |
| Self-assessment of the material situation | Very good | 1 | ref. |  |  |  |  |  |  |
|  | Good | 1.416 | 0.649 | 3..087 | 0.382 |  |  |  |  |
|  | Average/bad | 1.932 | 0.875 | 4.264 | 0.103 |  |  |  |  |
| White bread/rolls | Everyday | 1 | ref. |  |  |  |  |  |  |
|  | Rarely | 1.022 | 0.69 | 1.513 | 0.915 |  |  |  |  |
| Wholemeal bread | A few times a month or less | 1 | ref. |  |  |  |  |  |  |
|  | 1--4 times a week | 1.282 | 0.814 | 2.021 | 0.284 |  |  |  |  |
|  | Every day | 0.949 | 0.568 | 1.585 | 0.84 |  |  |  |  |
| Fishes and seafood | I don’t eat | 1 | ref. |  |  | 1 | ref. |  |  |
|  | A few times a month | 0.517 | 0.285 | 0.938 | 0.03 * | 0.514 | 0.269 | 0.984 | 0.045 * |
|  | Once a week or often | 0.61 | 0.33 | 1..127 | 0.115 | 0.638 | 0.327 | 1.244 | 0.187 |
| Red meat, ham, sausages | A few times a month or less | 1 | ref. |  |  |  |  |  |  |
|  | 1-4 times a week | 0.979 | 0.633 | 1.514 | 0.922 |  |  |  |  |
|  | Everyday | 0.996 | 0.575 | 1.727 | 0.99 |  |  |  |  |
| Sour milk products | A few times a month or less | 1 | ref. |  |  |  |  |  |  |
|  | 1-4 times a week | 0.954 | 0.583 | 1.56 | 0.851 |  |  |  |  |
|  | Everyday | 0.805 | 0.466 | 1.393 | 0.439 |  |  |  |  |
| Cheese | A few times a month or less | 1 | ref. |  |  |  |  |  |  |
|  | 1-4 times a week | 1.082 | 0.688 | 1.702 | 0.734 |  |  |  |  |
|  | Everyday | 0.934 | 0.559 | 1.56 | 0.793 |  |  |  |  |
| Cottage cheese | A few times a month or less | 1 | ref. |  |  |  |  |  |  |
|  | 1-4 times a week | 0,854 | 0,519 | 1,405 | 0,535 |  |  |  |  |
|  | Everyday | 1.335 | 0.734 | 2.427 | 0.344 |  |  |  |  |
| Vegetables/fruit | Everyday | 1 | ref. |  |  |  |  |  |  |
|  | Rarely | 1.107 | 0.723 | 1.695 | 0.64 |  |  |  |  |
| Sweets/salty snacks | A few times a month or less | 1 | ref. |  |  |  |  |  |  |
|  | 1-4 times a week | 0.802 | 0.476 | 1.35 | 0.406 |  |  |  |  |
|  | Everyday | 0.706 | 0.394 | 1.264 | 0.241 |  |  |  |  |
| Fast food products | I don’t eat | 1 | ref. |  |  | 1 | ref. |  |  |
|  | Consume | 0.584 | 0.391 | 0.87 | 0.008 * | 0.59 | 0.374 | 0.931 | 0.023 * |
| Body Mass Index (BMI) | Normal or underweight | 1 | ref. |  |  | 1 | ref. |  |  |
|  | Overweight | 1.365 | 0.862 | 2.161 | 0.185 | 0.797 | 0.43 | 1.477 | 0.471 |
|  | Class I obesity | 2.595 | 1.465 | 4.597 | 0.001 * | 0.875 | 0.352 | 2.176 | 0.774 |
|  | Class II and III obesity | 1.891 | 0.894 | 4.001 | 0.096 | 0.529 | 0.129 | 2.17 | 0.377 |
| Waist Hip Ratio (WHR) | Normal | 1 | ref. |  |  | 1 | ref. |  |  |
|  | Abdominal obesity | 2.354 | 1.58 | 3.508 | <0.001 * | 1.748 | 1.01 | 3.024 | 0.046 * |
| Waist to Height Ratio (WHtR) | Normal | 1 | ref. |  |  | 1 | ref. |  |  |
|  | Increased cardiometabolic risk | 2.078 | 1.35 | 3.2 | 0.001 * | 1.101 | 0.569 | 2.133 | 0.775 |
|  | Significantly increased cardiometabolic risk | 2,392 | 1,326 | 4,316 | 0,004 * | 0,761 | 0,259 | 2,238 | 0,62 |
| Systolic Blood Pressure | [mmHg] | 1.012 | 1.001 | 1.023 | 0.035 * | 0.991 | 0.973 | 1.009 | 0.341 |
| Blood Pressure | Normal | 1 | ref. |  |  | 1 | ref. |  |  |
|  | Elevated | 1.627 | 0.932 | 2.84 | 0.087 | 1.665 | 0.849 | 3.267 | 0..138 |
|  | High blood pressure Stage 1 | 2.405 | 1.503 | 3.848 | <0.001 * | 2.632 | 1.275 | 5.432 | 0.009 * |
|  | High blood pressure Stage 2 | 6.328 | 2.617 | 15.302 | <0.001 * | 7.096 | 1.886 | 26.703 | 0.004 * |
| Fasting glucose | Normal | 1 | ref. |  |  |  |  |  |  |
|  | Abnormal | 1.002 | 0.661 | 1.52 | 0.992 |  |  |  |  |
| Body Fat Percentage (BFP) category | Normal | 1 | ref. |  |  |  |  |  |  |
|  | Elevated | 1.484 | 0.957 | 2.302 | 0.078 |  |  |  |  |
|  | Excessive | 3.435 | 1.732 | 6.813 | <0.001 * |  |  |  |  |
| Visceral Fat Index | Normal | 1 | ref. |  |  | 1 | ref. |  |  |
|  | Elevated | 2.8 | 0.989 | 7.925 | 0.052 | 1.333 | 0.36 | 4.941 | 0.667 |
| Total Body Water (TBW) | Normal/hight, | 1 | ref. |  |  | 1 | ref. |  |  |
|  | Low | 2.299 | 1.449 | 3.649 | <0.001 * | 1.443 | 0.713 | 2.92 | 0.308 |
| Phase angle | | 1.066 | 0.719 | 1.579 | 0.752 |  |  |  |  |

* Statistically significant relationship (p<0.05); OR - odds ratio; CI – confidence interval; OR (95% CI) - odds ratio with a 95% confidence interval.
