# Supplementary material for: Prevalence of dyslipidaemia within Polish nurses. Cross-sectional study - single and multiple linear regression models and ROC analysis
Source: BMC Public Health. 2024 Apr 10;24:1002. doi: 10.1186/s12889-024-18542-6 (PMC11008020; doi:10.1186/s12889-024-18542-6)
Supplement: Supplementary file 2 — Supplementary Material 2 [file 12889_2024_18542_MOESM2_ESM.docx]

Table S1 presents single and multiple logistic regression models indicating significant (p < 0.05) predictors of the risk of developing hypercholesterolemia in the study group. Single-factor logistic regression models (separate for each of the analyzed features) showed that significant predictors (p˂0.05) of the risk of developing hypercholesterolemia are age (OR=.058), participation in preventive examinations other than obligatory (OR=1.834), obesity and BMI grade (OR=3.096), WHR abdominal obesity (OR=2.135), WHtR increased cardiometabolic risk (OR=1.969), WHtR significantly increased cardiometabolic risk (OR=2.117), SBP (OR=1.012), hypertension Stage I (OR=1.888), stage II hypertension (OR=15.573), increased body fat (OR=1.641), excessive body fat (OR=3.199), low body water (OR=2.085). The multiple logistic regression model showed that age (OR=1.038) and grade II hypertension (OR=12.589) are significant (p˂0.05) independent predictors of the risk of developing hypercholesterolemia.

**Table S1. Risk factors for development of hypercholesterolemia. Single and multiple analysis.**

| **Variable** | | **Univariate model** | | | | **Multiple model** | | | |
| --- | --- | --- | --- | --- | --- | --- | --- | --- | --- |
|  |  | **OR** | **95%CI** | | ***p*** | **OR** | **95%CI** | | ***p*** |
| Sex | Female | 1 | ref. |  |  |  |  |  |  |
|  | Male | 0.469 | 0.207 | 1.061 | 0.069 |  |  |  |  |
| Age | [years] | 1.058 | 1.036 | 1.08 | <0.001 * | 1.038 | 1.013 | 1.064 | 0.003 * |
| Place of residence | City | 1 | ref. |  |  |  |  |  |  |
|  | Village | 1.11 | 0.743 | 1.657 | 0.61 |  |  |  |  |
| Type of job | Staff management/administration | 1 | ref. |  |  |  |  |  |  |
|  | Hospital ward | 1.265 | 0.726 | 2.203 | 0.407 |  |  |  |  |
| Work system | One shift work (8h) | 1 | ref. |  |  |  |  |  |  |
|  | Shift work and night duty (12h) | 1.051 | 0.69 | 1.599 | 0.818 |  |  |  |  |
| More than one job | No | 1 | ref. |  |  |  |  |  |  |
|  | Yes | 1.106 | 0.735 | 1.662 | 0.63 |  |  |  |  |
| Education | Basic nursing education | 1 | ref. |  |  |  |  |  |  |
|  | Bachelor | 0.838 | 0.481 | 1.458 | 0.531 |  |  |  |  |
|  | Master degree | 0.633 | 0.396 | 1.012 | 0.056 |  |  |  |  |
| Participation in preventive examinations other than obligatory | No | 1 | ref. |  |  | 1 | ref. |  |  |
|  | Yes | 1.834 | 1.145 | 2.938 | 0.012 * | 1.51 | 0.909 | 2.509 | 0.112 |
| Cigarettes smoking | No | 1 | ref. |  |  |  |  |  |  |
|  | Yes | 1.221 | 0.763 | 1.955 | 0.405 |  |  |  |  |
| Adding sugar to coffe/tea | Nie | 1 | ref. |  |  |  |  |  |  |
|  | Tak | 1.439 | 0.961 | 2.155 | 0.077 |  |  |  |  |
| Salting dishes | Rarely or never add salt to food | 1 | ref. |  |  |  |  |  |  |
|  | I taste the food and add salt as needed | 1.04 | 0.615 | 1.759 | 0.883 |  |  |  |  |
|  | I add salt to my food without trying it first | 1.246 | 0.669 | 2.32 | 0.489 |  |  |  |  |
| Weight self-control* | Every day | 1 | ref. |  |  |  |  |  |  |
|  | Twice a week | 1.186 | 0.556 | 2.533 | 0.659 |  |  |  |  |
|  | Once a month | 0.898 | 0.494 | 1.631 | 0.724 |  |  |  |  |
|  | Hardly ever | 0.851 | 0.446 | 1.623 | 0.625 |  |  |  |  |
|  | I do not check my weight regulary | 0.82 | 0.454 | 1.483 | 0.511 |  |  |  |  |
| Self-assessment of the material situation | Very good | 1 | ref. |  |  |  |  |  |  |
|  | Good | 1.372 | 0.636 | 2.962 | 0.421 |  |  |  |  |
|  | Average/Bad | 1.52 | 0.695 | 3.328 | 0.294 |  |  |  |  |
| White bread/rolls | Every day | 1 | ref. |  |  |  |  |  |  |
|  | Less often | 0.887 | 0.593 | 1.328 | 0.561 |  |  |  |  |
| Wholemeal bread | A few times a month or less | 1 | ref. |  |  |  |  |  |  |
|  | 1-4 times a week | 1.111 | 0.694 | 1.78 | 0.66 |  |  |  |  |
|  | Every day | 0.752 | 0.447 | 1.267 | 0.285 |  |  |  |  |
| Fishes and seafood | I don’t eat | 1 | ref. |  |  |  |  |  |  |
|  | A few times a month or less | 0.609 | 0.324 | 1.144 | 0.123 |  |  |  |  |
|  | Once a week or often | 0.559 | 0.292 | 1.067 | 0.078 |  |  |  |  |
| Red meat, ham, sausages | A few times a month or less | 1 | ref. |  |  |  |  |  |  |
|  | 1-4 times a week | 0.975 | 0.625 | 1.522 | 0.912 |  |  |  |  |
|  | Every day | 1.397 | 0.783 | 2.495 | 0.258 |  |  |  |  |
| Sour milk products | A few times a month or less | 1 | ref. |  |  |  |  |  |  |
|  | 1-4 times a week | 0.807 | 0,481 | 1,353 | 0,416 |  |  |  |  |
|  | Every day | 0.62 | 0.351 | 1.094 | 0.099 |  |  |  |  |
| Cheese | A few times a month or less | 1 | ref. |  |  |  |  |  |  |
|  | 1-4 times a week | 0.953 | 0.6 | 1.514 | 0.839 |  |  |  |  |
|  | Every day | 1.171 | 0.687 | 1.994 | 0.562 |  |  |  |  |
| Cottage cheese | A few times a month or less | 1 | ref. |  |  |  |  |  |  |
|  | 1-4 times a week | 0.761 | 0.454 | 1.276 | 0.301 |  |  |  |  |
|  | Every day | 1.091 | 0.584 | 2.037 | 0.785 |  |  |  |  |
| Vegetables/fruit | Everyday | 1 | ref. |  |  |  |  |  |  |
|  | Rarely | 1.261 | 0.81 | 1.965 | 0.304 |  |  |  |  |
| Sweets/salty snacks | A few times a month or less | 1 | ref. |  |  |  |  |  |  |
|  | 1-4 times a week | 0.741 | 0.43 | 1.277 | 0.281 |  |  |  |  |
|  | Every day | 0.791 | 0.431 | 1.45 | 0.448 |  |  |  |  |
| Fast food products | I don’t eat | 1 | ref. |  |  |  |  |  |  |
|  | Consume | 0.781 | 0.519 | 1.176 | 0.237 |  |  |  |  |
| Body Mass Index (BMI) | Normal or underweight | 1 | ref. |  |  | 1 | ref. |  |  |
|  | Overweight | 1.273 | 0.801 | 2.023 | 0.308 | 0.891 | 0.487 | 1.63 | 0.707 |
|  | Class I obesity | 3.096 | 1.645 | 5.827 | <0.001 * | 1.67 | 0.636 | 4.388 | 0.298 |
|  | Class II or III obesity | 1.785 | 0.818 | 3.899 | 0.146 | 1.139 | 0.261 | 4.962 | 0.863 |
| Waist Hip Ratio (WHR) | Normal | 1 | ref. |  |  | 1 | ref. |  |  |
|  | Abdominal obesity | 2.135 | 1.417 | 3.217 | <0.001 * | 1.511 | 0.862 | 2.648 | 0.15 |
| Waist to Height Ratio (WHtR) | Normal | 1 | ref. |  |  | 1 | ref. |  |  |
|  | Increased cardiometabolic risk | 1.969 | 1.266 | 3.06 | 0.003 * | 0.99 | 0.508 | 1.929 | 0.976 |
|  | Significantly increased cardiometabolic risk | 2.117 | 1.146 | 3.908 | 0.017 * | 0.475 | 0.153 | 1.477 | 0.198 |
| Systolic Blood Pressure (SBP) | [mmHg] | 1.012 | 1 | 1.023 | 0.044 * | 0.993 | 0.978 | 1.008 | 0.369 |
| Blood Pressure | Normal | 1 | ref. |  |  | 1 | ref. |  |  |
|  | Elevated | 1.449 | 0.827 | 2.542 | 0.195 | 1.182 | 0.616 | 2.267 | 0.615 |
|  | High blood pressure Stage 1 | 1.888 | 1.173 | 3.037 | 0.009 * | 1.676 | 0.859 | 3.268 | 0.13 |
|  | High blood pressure Stage 2 | 15.573 | 3.624 | 66.928 | <0.001 * | 12.589 | 2.342 | 67.679 | 0.003 * |
| Fasting glucose | Normal | 1 | ref. |  |  |  |  |  |  |
|  | Abnormal | 1.022 | 0.666 | 1.568 | 0.92 |  |  |  |  |
| Body Fat Percentage (BFP) category | Normal | 1 | ref. |  |  |  |  |  |  |
|  | Elevated | 1.641 | 1.041 | 2.586 | 0.033 * |  |  |  |  |
|  | Excessive | 3.199 | 1.525 | 6.713 | 0.002 * |  |  |  |  |
| Visceral fat index | Normal | 1 | ref. |  |  | 1 | ref. |  |  |
|  | Elevated | 2.436 | 0.793 | 7.478 | 0.12 | 0.911 | 0.218 | 3.813 | 0.899 |
| Total Body Water (TBW) | Normal/High | 1 | ref. |  |  | 1 | ref. |  |  |
|  | Low | 2.085 | 1.279 | 3.4 | 0.003 * | 1.061 | 0.505 | 2.23 | 0.876 |
| Phase angle | | 0.918 | 0.613 | 1.373 | 0.677 |  |  |  |  |

* Statistically significant relationship (p<0.05); OR - odds ratio; CI – confidence interval; OR (95% CI) - odds ratio with a 95% confidence interval.
