# Supplementary material for: Prevalence of dyslipidaemia within Polish nurses. Cross-sectional study - single and multiple linear regression models and ROC analysis
Source: BMC Public Health. 2024 Apr 10;24:1002. doi: 10.1186/s12889-024-18542-6 (PMC11008020; doi:10.1186/s12889-024-18542-6)
Supplement: Supplementary file 3 — Supplementary Material 3 [file 12889_2024_18542_MOESM3_ESM.docx]

Table S3 presents single and multiple logistic regression models indicating significant (p < 0.05) predictors of the risk of developing elevated total cholesterol (TC) in the study group. Single logistic regression models (separate for each of the analyzed characteristics) showed that significant (p˂0.05) predictors of the risk of developing elevated TC are: male gender (OR=0.363), age (OR=1.061), participation in preventive examinations other than obligatory (OR=1.855), 1st degree obesity according to BMI (OR=2.595), abdominal obesity according to WHR (OR=2.047), increased cardiometabolic risk according to WHtR (OR=1.895), SBP (OR=1.012), 1st degree hypertension ( OR=1.864), grade II hypertension (OR=11.043), excessive body fat (OR=2.352) and low body water (OR=1.853). The multiple logistic regression model showed that significant (p˂0.05) independent predictors of the risk of elevated TC are male gender (OR=0.279), age (OR=1.042) and grade II hypertension: OR=11.487).

**Table S3. Risk factors for increased total cholesterol in the study group. Single and multiple analysis.**

| **Variable** | | **Univariate model** | | | | **Multiple model** | | | |
| --- | --- | --- | --- | --- | --- | --- | --- | --- | --- |
|  |  | **OR** | **95%CI** | | ***p*** | **OR** | **95%CI** | | ***p*** |
| Sex | Female | 1 | ref. |  |  | 1 | ref. |  |  |
|  | Male | 0.363 | 0.156 | 0.842 | 0.018 * | 0.279 | 0.095 | 0.818 | 0.02 * |
| Age | [years] | 1.061 | 1.039 | 1.083 | <0.001 * | 1.042 | 1.016 | 1.068 | 0.001 * |
| Place of residence | City | 1 | ref. |  |  |  |  |  |  |
|  | Village | 1.124 | 0.756 | 1.672 | 0.564 |  |  |  |  |
| Type of work | Staff management/administration | 1 | ref. |  |  |  |  |  |  |
|  | Hospital ward | 1.328 | 0.766 | 2.305 | 0.312 |  |  |  |  |
| Work system | One shift work [8h] | 1 | ref. |  |  |  |  |  |  |
|  | Shift work and night duty [12h] | 1.044 | 0.689 | 1.584 | 0.838 |  |  |  |  |
| More than one job | No | 1 | ref. |  |  |  |  |  |  |
|  | Yes | 1.137 | 0.759 | 1.703 | 0.533 |  |  |  |  |
| Education | Basic nursing education | 1 | ref. |  |  |  |  |  |  |
|  | Bachelor | 0.839 | 0.487 | 1.446 | 0.528 |  |  |  |  |
|  | Master degree | 0.692 | 0.436 | 1.1 | 0.12 |  |  |  |  |
| Participation in preventive examinations other than obligatory | No | 1 | ref. |  |  | 1 | ref. |  |  |
|  | Yes | 1.855 | 1.166 | 2.95 | 0.009 * | 1.452 | 0.877 | 2.406 | 0.147 |
| Cigarettes smoking | No | 1 | ref. |  |  |  |  |  |  |
|  | Yes | 1.101 | 0.694 | 1.747 | 0.683 |  |  |  |  |
| Adding sugar to coffe/tea | No | 1 | ref. |  |  |  |  |  |  |
|  | Yes | 1.322 | 0.886 | 1.971 | 0.172 |  |  |  |  |
| Salting dishes | Rarely or never add salt to food | 1 | ref. |  |  |  |  |  |  |
|  | I taste the food and add salt as needed | 1.059 | 0.629 | 1.783 | 0.828 |  |  |  |  |
|  | I add salt to my food without trying it first | 1.264 | 0.683 | 2.339 | 0.455 |  |  |  |  |
| Weight self-control* | Once a week lub rzadziej | 1 | ref. |  |  |  |  |  |  |
|  | Twice a week | 1.312 | 0.623 | 2.764 | 0.474 |  |  |  |  |
|  | Once a month | 1.05 | 0.583 | 1.892 | 0.871 |  |  |  |  |
|  | Hardly ever | 0.926 | 0.49 | 1.748 | 0.812 |  |  |  |  |
|  | I do not check my weight regulary | 0.96 | 0.536 | 1.722 | 0.892 |  |  |  |  |
| Self-assessment of the material situation | Very good | 1 | ref. |  |  |  |  |  |  |
|  | Good | 1.447 | 0.672 | 3.117 | 0.345 |  |  |  |  |
|  | Average/Bad | 1.569 | 0.719 | 3.424 | 0.258 |  |  |  |  |
| White bread/rolls | Every day | 1 | ref. |  |  |  |  |  |  |
|  | Rarely | 0.816 | 0.548 | 1.217 | 0.319 |  |  |  |  |
| Wholemeal bread | A few times a month or less | 1 | ref. |  |  |  |  |  |  |
|  | 1-4 times a week | 1.014 | 0.637 | 1.614 | 0.952 |  |  |  |  |
|  | Everyday | 0.77 | 0.458 | 1.294 | 0.324 |  |  |  |  |
| Fishes and seafood | I don’t eat | 1 | ref. |  |  |  |  |  |  |
|  | A few times a month | 0.641 | 0.347 | 1.185 | 0.156 |  |  |  |  |
|  | Once a week or often | 0.62 | 0.33 | 1.167 | 0.138 |  |  |  |  |
| Red meat, ham, sausages | A few times a month or less | 1 | ref. |  |  |  |  |  |  |
|  | 1-4 times a week | 0.997 | 0.641 | 1.55 | 0.989 |  |  |  |  |
|  | Everyday | 1.476 | 0.832 | 2.62 | 0.183 |  |  |  |  |
| Sour milk products | A few times a month or less | 1 | ref. |  |  |  |  |  |  |
|  | 1-4 times a week | 0.798 | 0.479 | 1.329 | 0.386 |  |  |  |  |
|  | Everyday | 0.635 | 0.362 | 1.114 | 0.113 |  |  |  |  |
| Cheese | A few times a month or less | 1 | ref. |  |  |  |  |  |  |
|  | 1-4 times a week | 0.946 | 0.598 | 1.498 | 0.814 |  |  |  |  |
|  | Everyday | 1.179 | 0.696 | 1.997 | 0.54 |  |  |  |  |
| Cottage cheese | A few times a month or less | 1 | ref. |  |  |  |  |  |  |
|  | 1-4 times a week | 0.758 | 0.455 | 1.262 | 0.286 |  |  |  |  |
|  | Everyday | 1.149 | 0.62 | 2.131 | 0.659 |  |  |  |  |
| Vegetables/fruit | Everyday | 1 | ref. |  |  |  |  |  |  |
|  | Niecodziennie | 1.236 | 0.798 | 1.914 | 0.343 |  |  |  |  |
| Sweets/salty snacks | A few times a month or less | 1 | ref. |  |  |  |  |  |  |
|  | 1-4 times a week | 0.859 | 0.505 | 1.459 | 0.573 |  |  |  |  |
|  | Everyday | 0.952 | 0.526 | 1.724 | 0.87 |  |  |  |  |
| Fast food products | I don’t eat | 1 | ref. |  |  |  |  |  |  |
|  | Consume | 0.829 | 0.553 | 1.241 | 0.362 |  |  |  |  |
| Body Mass Index (BMI) | Normal/underweight | 1 | ref. |  |  | 1 | ref. |  |  |
|  | Overweight | 1.295 | 0.816 | 2.056 | 0.273 | 0.902 | 0.489 | 1.664 | 0.741 |
|  | Class I obesity | 2.595 | 1.418 | 4.75 | 0.002 * | 1.172 | 0.451 | 3.042 | 0.745 |
|  | Class II and III obesity | 1.281 | 0.606 | 2.707 | 0.517 | 0.584 | 0.139 | 2.451 | 0.462 |
| Waist Hip Ratio (WHR) | Normal | 1 | ref. |  |  | 1 | ref. |  |  |
|  | Abdominal obesity | 2.047 | 1.366 | 3.067 | 0.001 * | 1.345 | 0.751 | 2.407 | 0.318 |
| Waist to Height Ratio (WHtR) | Normal | 1 | ref. |  |  | 1 | ref. |  |  |
|  | Increased cardiometabolic risk | 1.895 | 1.223 | 2.935 | 0.004 * | 1.122 | 0.559 | 2.251 | 0.746 |
|  | Significantly increased cardiometabolic risk | 1.663 | 0.921 | 3.003 | 0.092 | 0.513 | 0.165 | 1.589 | 0.247 |
| Systolic Blood Pressure | [mmHg] | 1.012 | 1 | 1.023 | 0.044 * | 0.995 | 0.981 | 1.01 | 0.519 |
| Blood Pressure | Normal | 1 | ref. |  |  | 1 | ref. |  |  |
|  | Elevated | 1.499 | 0.857 | 2.623 | 0.156 | 1.409 | 0.722 | 2.751 | 0.315 |
|  | High blood pressure Stage 1 | 1.864 | 1.164 | 2.984 | 0.01 * | 1.777 | 0.914 | 3.458 | 0.09 |
|  | High blood pressure Stage 2 | 11.043 | 3.26 | 37.406 | <0.001 * | 11.487 | 2.544 | 51.871 | 0.002 * |
| Fasting glucose | Normal | 1 | ref. |  |  |  |  |  |  |
|  | Abnormal | 1.017 | 0.666 | 1.555 | 0.936 |  |  |  |  |
| Body Fat Percentage (BFP) category | Normal | 1 | ref. |  |  |  |  |  |  |
|  | Elevated | 1.472 | 0.94 | 2.305 | 0.091 |  |  |  |  |
|  | Excessive | 2.352 | 1.186 | 4.663 | 0.014 * |  |  |  |  |
| Visceral Fat Index (VFI) | Normal | 1 | ref. |  |  | 1 | ref. |  |  |
|  | Elevated | 1.982 | 0.7 | 5.614 | 0.198 | 1.557 | 0.379 | 6.4 | 0.539 |
| Total Body Water (TBW) | Normal/High | 1 | ref. |  |  | 1 | ref. |  |  |
|  | Low | 1.853 | 1.154 | 2,974 | 0.011 * | 1.098 | 0.528 | 2.285 | 0.802 |
| Phase angle | | 0.848 | 0.569 | 1,264 | 0.418 |  |  |  |  |

* Statistically significant relationship (p<0.05); OR - odds ratio; CI – confidence interval; OR (95% CI) - odds ratio with a 95% confidence interval
